# Spinal Arachnoiditis in Patients with Coccidioidomycosis Meningitis—Analysis of Clinical and Imaging Features

**DOI:** 10.3390/jof8111180

**Published:** 2022-11-08

**Authors:** Geetha Sivasubramanian, Saurin Kadakia, Jani M. Kim, Sarah Pervaiz, Yueqi Yan, Robert Libke

**Affiliations:** 1 Department of Infectious Disease, University of California, Fresno, CA 93701, USA; 2 Department of Internal Medicine, University of California, Fresno, CA 93701, USA; 3 HSRI Biostatistics and Data Support Core, University of California, Merced, CA 95343, USA

**Keywords:** coccidioidomycosis meningitis, arachnoiditis, syringomyelia

## Abstract

**Background:** Coccidioidomycosis meningitis (CM) is the most aggressive form of coccidioidomycosis, requiring lifelong antifungal treatment and often cerebrospinal fluid (CSF) diversion. Long-standing CM can be associated with spinal complications such as arachnoiditis. However, studies describing the frequency, clinical, and imaging characteristics of arachnoiditis in patients with CM are limited. **Methods:** We identified 133 patients with CM based on CSF culture, PCR, or serology between January 2010 and December 2020. Of these, 37 patients underwent spinal imaging. Data on demographics, risk factors, symptoms, antifungal therapy, surgical management, follow-up visits, adherence, serological trends, and imaging findings were reviewed. **Results:** Abnormal findings were observed in 30 of the 37 patients with CM who underwent spinal imaging. The imaging abnormalities noted in our study included leptomeningeal enhancement (53%), arachnoiditis (53%), syringomyelia (23%), cord signal abnormalities (10%), and osteomyelitis (7%). Of the 30 patients, 90% had symptoms, such as weakness, numbness, or urinary retention. The incidence of arachnoiditis in the present study was 12%. Higher initial CSF protein levels and intra cranial pressure were associated with a higher risk of developing arachnoiditis/syringomyelia. Management of CM was challenging, as evidenced by shunt failure (46%), medication non-compliance (57%), and lack of adequate follow-up (60%). Persistent disabilities were noted in 62% of the patients. **Conclusions:** Patients with CM develop spinal complications such as arachnoiditis, or syringomyelia. Many cases may go undetected due to lack of symptoms in early stages. CM management challenges such as shunt failure, lack of follow-up care, and medication noncompliance, were frequent.

## 1. Introduction

Coccidioidomycosis is a fungal infection caused by the dimorphic fungi Coccidioides immitis and Coccidioides posadasii [1]. The fungus is endemic to southwestern United States, especially California and Arizona. The number of annual cases has increased over the last 10 years [2]. Fungal mycelia in the soil produce arthroconidia that are aerosolized and inhaled. The inhaled arthroconidia transform into spherules in the lung tissue, which mature and release the endospores. Endospores, in addition to local tissue inflammation, can disseminate to distant organs causing disseminated disease [3]. More than 60% of the cases are either asymptomatic or have mild self-limited respiratory infections. Dissemination occurs in less than 1–3% of the population to distant sites such as lymph nodes, bones, skin, and the central nervous system (CNS) [4]. Known risk factors for disseminated infections include age, ethnicity (Filipino and African American), uncontrolled diabetes, pregnancy, smoking, and immunocompromising conditions, such as Human Immunodeficiency virus (HIV) infection and transplantation [5].

Coccidioidomycosis meningitis (CM) is the most severe and potentially fatal form of this infection. Extensive inflammation of the leptomeninges, especially in the basilar portions, may result in a thick membrane formation obliterating the subarachnoid space, resulting in increased intracranial pressure and hydrocephalus [6]. Patients usually present with headache and altered mentation. Intracranial pressure management in addition to antifungal therapy is essential. Other complications of CM include vasculitis, arachnoiditis, and syringomyelia. Patients with CM often have inflammation of the cervical, thoracic, or lumbar spinal cord arachnoid membrane, leading to arachnoiditis [7]. Terms used to describe “spinal coccidioidomycosis” in the literature can often be confusing as this has been used interchangeably to describe changes related to arachnoiditis, a complication of CM as well as to refer to vertebral osteomyelitis or intra medullary infection from dissemination of primary infection to bone or spinal cord [8,9,10]. If spinal imaging is to be pursued in all patients with CM, abnormalities related to meningeal enhancement and arachnoiditis could be noted in many [11]. However, this has been systematically studied in very few studies [12,13]. We performed a retrospective study of patients with CM in our center between 2010 and 2020 and studied patients who underwent spinal imaging. Data on demographics, risk factors, symptoms, antifungal therapy, surgical management, follow-up visits, adherence, serological trends, and imaging findings were reviewed.

## 2. Methods

### 2.1. Patients Selection

Using a retrospective medical record review, we identified 133 patients with CM between January 2010 and December 2020 at our 700-bed tertiary referral center in Fresno, California. Patients with CM were identified using ICD 9 and ICD 10 codes. Individual charts were then reviewed to determine whether they met criteria for coccidioidal meningitis. CM was defined as either positive CSF polymerase chain reaction, cultures, or detection of complement-fixing antibodies to Coccidioides antigen in the CSF or typical CSF abnormalities with detection of serum complement-fixing antibodies with compatible clinical illness. Compatible clinical illnesses included fever, chills, headache, changes in mentation and ataxia. Typical CSF findings include pleocytosis (WBC > 5 per cubic millimeter), elevated protein, and/or reduced glucose levels. We then identified patients who underwent spinal imaging at any time after the diagnosis of CM to be included in this study.

### 2.2. Data Extraction

We reviewed the patient data from the initial diagnosis of CM and their follow-up visits. Baseline information was collected including demographics (age, ethnicity, race, and occupational exposure) and underlying medical conditions (diabetes, HIV, pregnancy, immunocompromising conditions, smoking, alcohol consumption, and drug use). Clinical, laboratory, and imaging data pertinent to CM, including clinical features, cultures, serology, PCR, brain imaging results, antifungal management, and intracranial pressure management, were collected, including follow-up visits, treatment course, serological trend, response to therapy, and outcomes. With regard to spinal imaging, the time interval since the diagnosis of CM, symptoms related to spinal involvement, spinal imaging findings, anti-fungal management, neurosurgical interventions, response to therapy, follow-up imaging, clinical course, and outcomes were reviewed.

### 2.3. Statistical Analysis

Chi-square (or Fisher’s exact test) and *t*-test (or nonparametric equality-of-medians test) were performed to compare demographic or clinical characteristics and outcomes between arachnoiditis negative and positive groups.

The study was approved by the institutional review board.

## 3. Results

We identified 133 patients with CM at our center between 2010 and 2020. Of 133 patients with CM, 37 who underwent spinal imaging after the diagnosis of CM were included in the study. The median age of the study population was 39 years, and 78% were male (Table 1). Approximately 50% of the study population had occupational outdoor exposure to coccidioidomycosis.

**Figure 1:**
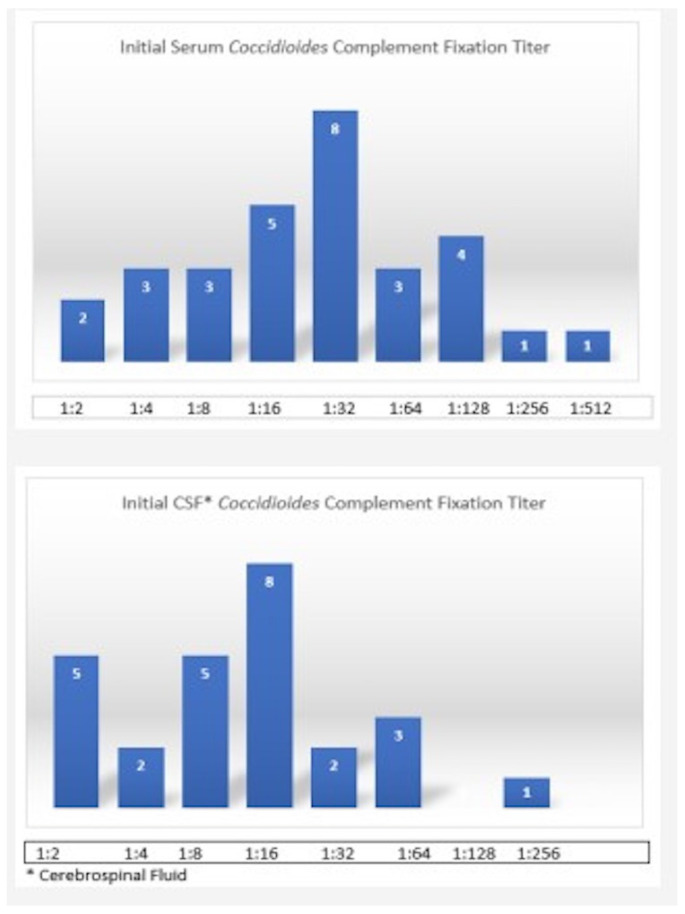
Serum and cerebrospinal fluid serological trends in study patients.

The initial serum coccidioidomycosis complement fixation titer ≥ 1:32 was 57% and the initial CSF coccidioidomycosis complement fixation titer ≥ 1:16 in 53% of patients (Figure 1). Intrathecal amphotericin was used for management in only one patient with abnormal spinal imaging, with fluconazole as the antifungal agent of choice. The median treatment duration was six years. The management of CM was challenging, as evidenced by shunt failure (62%), serological failure (51%), medication non-compliance (51%), and lack of adequate follow-up (48%) (Table 2). Patients were followed for a median of 48 months.

**Figure 2:**
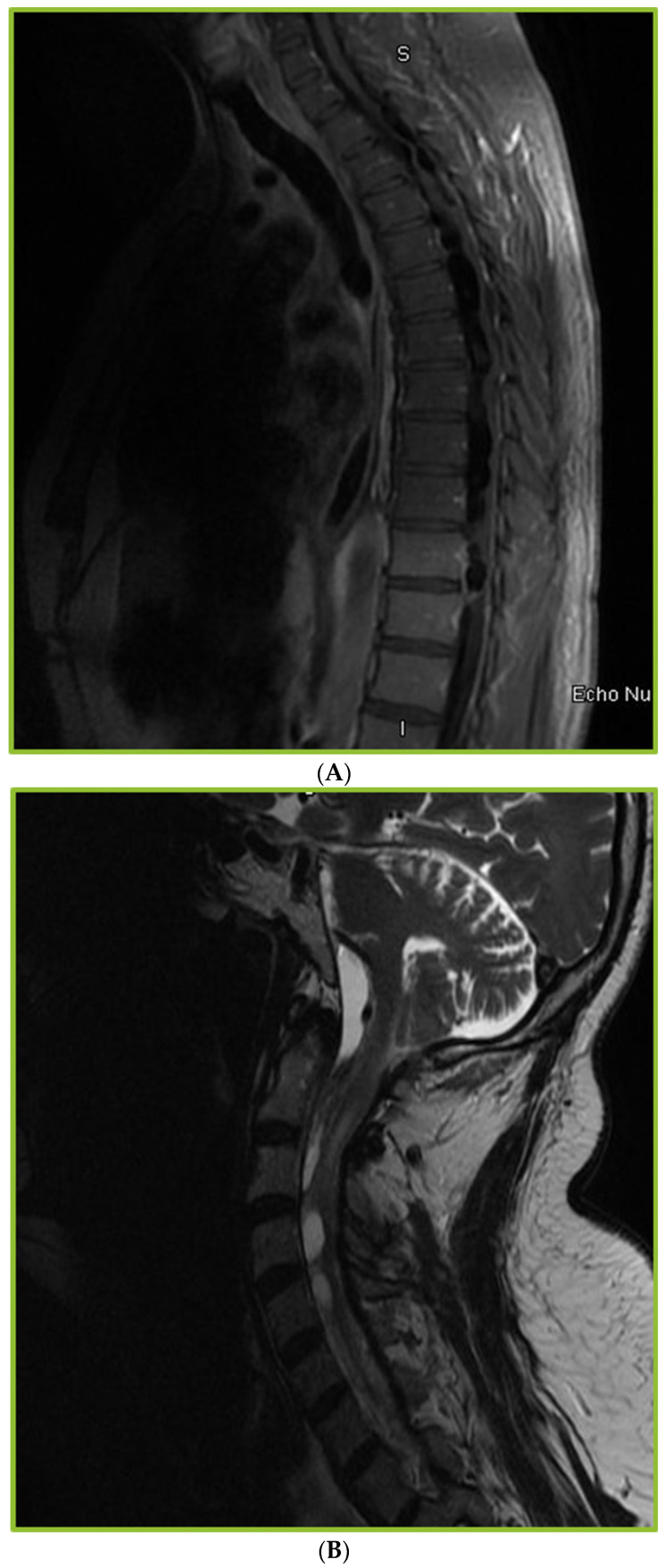

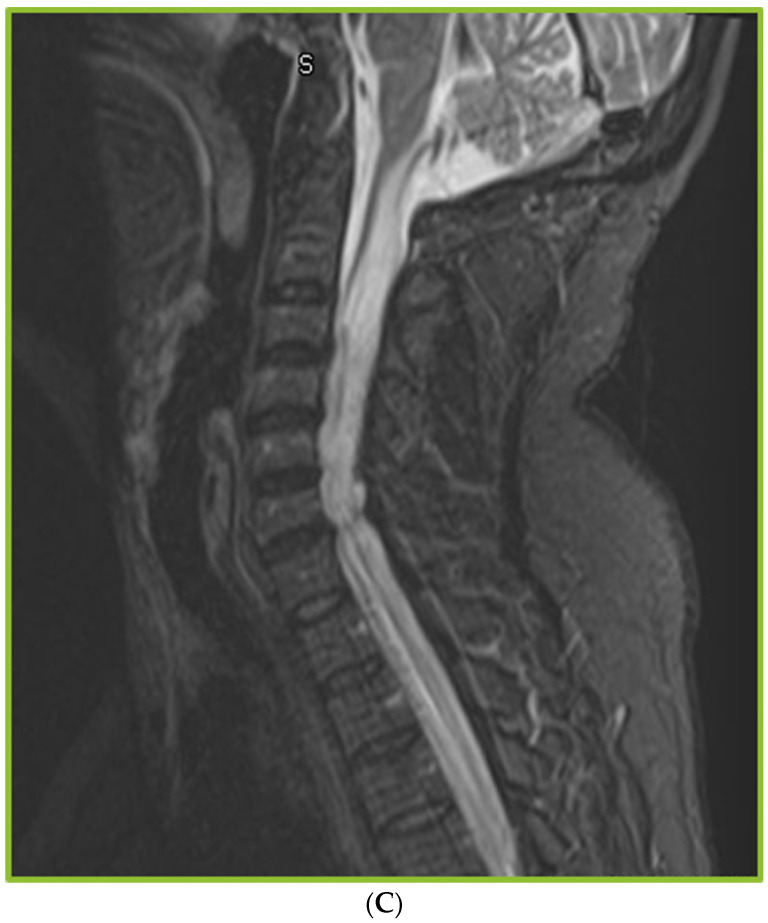
(**A**) Sagittal thoracic MRI spine: Septations, ill-defined irregularity in the intrathecal space, deformity of the thoracic cord, complex ill-defined CSF collections. (**B**) Sagittal post contrast MRI cervical spine: Extensive multilocular fluid tracking from the cervical medullary junction to C5 level with marginal rim enhancement. (**C**) Sagittal cervical and thoracic MRI spine: Extensive syringohydromyelia present throughout the entire cervical cord and upper thoracic cord.

**Figure 3:**
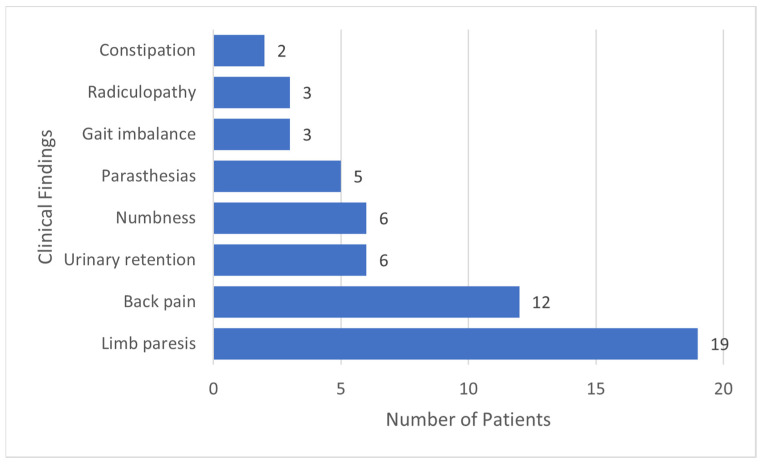
Frequency of symptoms secondary to spinal abnormalities in study patients.

Abnormal findings were observed in 80% of the patients with CM who underwent spinal imaging (30/37). The imaging abnormalities noted in our study included leptomeningeal enhancement (53%), arachnoiditis (53%), syringomyelia (23%), cord signal abnormalities (10%), and osteomyelitis (7%). The incidence of arachnoiditis and syringomyelia on spinal imaging was 12%. Findings of arachnoiditis included trapped CSF/tethering of the spinal cord/nerve root clumping in addition to the development of syringomyelia (Figure 2A–C). Of the 30 patients with abnormal findings, 90% had symptoms, such as weakness, numbness, or urinary retention (Figure 3). Most patients with arachnoiditis were managed with antifungal therapy alone. Only two patients received adjuvant corticosteroids. Surgical intervention for spinal complications, such as shunting of the cyst, duraplasty, and laminectomy, was performed in three patients. Persistent disabilities were present in 62% of the patients. Follow-up spinal imaging (37%) continued to show persistent spinal abnormalities. No statistically significant differences were found between the groups with negative (N= 20) and positive arachnoiditis and/or syringomyelia (N = 17) in most demographic and clinical characteristics, management, and outcomes. However, the positive arachnoiditis and/or syringomyelia group had significantly higher average initial CSF opening pressure (17.5 vs. 9.9, *p* = 0.034) and higher average CSF protein (877.9 vs. 255.6, *p* = 0.036) at the time of CM diagnosis, compared with the group with negative arachnoiditis and/or syringomyelia (Table 3). Spine MRI was performed at a median duration of 24 months after the diagnosis of CM. The positive arachnoiditis and/or syringomyelia group had a marginally significantly longer median duration between CM diagnosis and first spinal imaging than the negative group (36 vs. 13 months, *p* = 0.081).

## 4. Discussion

CM is a devastating form of coccidioidomycosis. The disease is insidious, progressive, and uniformly fatal if left untreated. Currently, anti-fungal management with lifelong azoles is the mainstay of therapy. CM has a predilection for the basilar meninges and causes fibrosis and inflammation in the subarachnoid space, resulting in ventricular dilatation and hydrocephalus. This often necessitates surgical management such as placement of a ventriculoperitoneal shunt [14]. While elevated intracranial pressure and hydrocephalus are well known complications of CM, there are other less studied complications such as vasculitis with strokes, arachnoiditis, and syringomyelia. Arachnoiditis refers to inflammation and scarring of the arachnoid mater, the central layer of membranes covering the spinal cord [15]. The ensuing fibrinous exudates and collagen adhesions cause the nerve roots to adhere and clump into the thecal sac. The lack of innervation and vasculature of the arachnoid membrane impedes healing. Persistent inflammation may lead to adhesive arachnoiditis, hyalinization, and calcification. Inflammation of the arachnoid membrane can lead to ischemia and vasculitis. Syringomyelia or spinal cord cyst formation is a complication of arachnoiditis when the scarring causes changes in the flow of CSF and/or the vascular supply, often resulting in paresis. The triggers for this inflammatory response may be chemical, mechanical, or infectious in nature [16]. Non-infectious triggers include prior spinal surgery, subarachnoid hemorrhage, epidural injections, and myelogram contrast agents [17]. Infections commonly associated with the development of spinal arachnoiditis include meningitis caused by *Mycobacterium tuberculosis* and fungi such as *Cryptococcus*, *Candida*, *Coccidioides*, and *Histoplasma*, as well as pathogens such as *Listeria* and *Cysticercus* [16,18,19,20]. Most literature on this topic relates to the development of this complication due to chronic meningitis caused by *Mycobacterium tuberculosis* [21,22,23]. The presentation of arachnoiditis is often insidious with variable severity and clinical manifestations. Several cases may be subclinical, asymptomatic, or undiagnosed. When present, the symptoms are due to nerve root compression and include back pain, urinary frequency, urgency, sensory loss, and weakness in the extremities. The symptoms related to arachnoiditis include back pain, radiculopathy, muscle weakness, and urinary retention [15]. MRI may reveal tethering, nerve root clumping, septations, and CSF fluid collections due to adhesions [12,13,24]. Studies describing the clinical features and outcomes of spinal arachnoiditis in patients with CM are limited. Two studies described radiological findings of the spine in patients with CM. In a study by Crete et al. describing MRI findings in 41 patients, diffuse leptomeningeal enhancement (63%) was the most common finding, followed by arachnoiditis/adhesive changes (54%), osteomyelitis (34%), cord edema (27%), and syrinx (7%) [13]. Another study by Lammering et al. showed a high prevalence of concurrent brain and spinal cord imaging abnormalities in patients with *Coccidioides* infection [12]. In this study of 23 patients with CM, 86% who underwent spinal imaging showed abnormalities, such as leptomeningeal enhancement (84%), arachnoiditis (63%), and cord signal changes (37%). Current clinical guidelines do not recommend routine spinal imaging in asymptomatic patients with CM [25]. Management is with aggressive antifungal therapy for symptomatic patients, possibly with adjuvant corticosteroids and surgical intervention if possible and necessary [26].

Of a large cohort of 133 patients with CM, we identified 37 who underwent spine imaging. Of the 37 patients who underwent spine imaging in our study, 30 (81%) were noted to have abnormalities. Incidence of abnormal spinal imaging findings in CM patients in our study was 23% and specifically of arachnoiditis was 12%. It is possible that the true incidence is higher, as there were likely more patients who had underlying changes of arachnoiditis but did not undergo spinal imaging due to lack of symptoms. The imaging abnormalities predominantly observed were leptomeningeal enhancement, arachnoiditis, and syringomyelia. Notably, concurrent findings of *Coccidioides* vertebral osteomyelitis were observed in only two patients in the study. Leptomeningeal enhancement often was diffuse across the entire spine. Arachnoiditis was observed more frequently in the cervical and thoracic spine than in the lumbar spine in our study. Syrinx formation was only observed in the cervical and thoracic cords. A total of 37 patients underwent follow-up spinal imaging, and as expected, persistent abnormalities were found in most patients. A previous study comparing the CM cohort from 2008 to 1980 found that lumbar arachnoiditis was seen in 10% patients in 2008 cohort as opposed to the 1980 cohort which had 35% incidence of arachnoiditis [27]. The higher incidence in the cohort of 1980 patients was felt to be secondary to more common use of intrathecal amphotericin B. Prior to widespread use of azoles for management of CM, intrathecal amphotericin B was used more frequently and considered a potential trigger of arachnoiditis [28]. Only one patient with abnormal spinal imaging had previously received intrathecal amphotericin B. The median duration between the initial CM diagnosis and first spinal imaging was two years. However, imaging was often performed as part of routine care in response to underlying symptoms. Only prospective studies of patients with CM with routine spinal imaging can identify the duration when spinal imaging abnormalities may be observed after the diagnosis of CM. Of the 30 patients with abnormal spinal imaging, 28 (93%) had symptoms. Limb paresis and back pain were the most common symptoms. Studies on the management of arachnoiditis and syringomyelia associated with CM are very limited [16]. Aggressive anti-fungal management and symptom control for pain relief are the main strategies with unclear roles for surgery and adjuvant steroids. Only three patients in our study underwent surgical procedures of the spine, such as cyst drainage and duraplasty. Follow-up imaging, when performed, showed improvement in CSF collection after shunting procedures; however, tethering and scarring persisted. Two patients received adjuvant corticosteroids with a modest improvement in pain. Further studies are needed to elucidate the role of surgery and corticosteroids in symptomatic patients with arachnoiditis.

Management of underlying CM was very challenging in our study population, with a high incidence of shunt failure (46%) requiring multiple readmissions and revision surgeries, anti-fungal medication non-compliance (57%), and lack of adequate follow-up (60%). Previous reports have alluded to medication non-compliance resulting in spinal complications in patients with CM [9]. However, no statistically significant differences were noted in our study regarding the underlying demographics, risk factors, management, or follow-up between those who developed arachnoiditis and syringomyelia and those who did not. High cerebrospinal fluid protein content is considered a risk factor for the development of arachnoiditis in patients with tubercular meningitis [23]. This was also noted in our study: elevated initial CSF protein concentration and elevated initial intracranial pressure were both noted to be higher risk factors for developing arachnoiditis or syringomyelia. Our study was limited by its retrospective nature and lack of spinal imaging data in the remaining patients with CM who did not develop spinal symptoms. There may be a role for early spinal imaging in all patients with CM which could guide more aggressive management of underlying CM and prevent further complications. However, another limiting factor in the care of these patients would be obtaining approval for spinal imaging from insurance companies in minimally symptomatic patients. A prospective study of patients with CM with close follow-up of spinal symptoms and serial spinal imaging may offer more insights to risk factors for development of arachnoiditis and potential prevention strategies. Significant and persistent disabilities due to development of arachnoiditis further complicates the management and care of individuals already dealing with a devastating entity in the form of *Coccidioidal* meningitis.

## Figures and Tables

**Table 1:** Characteristics of Patients with CM * (N = 37).

**Demographics**
Age: 39 [25, 55] (Median IQR **) Sex: Male 29 (78%), Female 8 (22%) Ethnicity: Hispanic 17 (46%), non-Hispanic 20 (54%) Race: Caucasian 24 (64%), African American 5 (13%), Asian 7(18%)
**Comorbidities and Risk factors**
Diabetes mellitus: 7 (18%) Human Immunodeficiency Virus: 4 (10%) Cancer: 2 (5%) Smoking: 12 (32%) Alcohol abuse: 7 (18%) Substance abuse: 7 (18%) Outdoor occupational exposure: 18 (48%)
**Serology**
Initial serum coccidioidomycosis complement fixation titer ≥ 1:32 in 57% Initial CSF *** coccidioidomycosis complement fixation titer ≥ 1:16 in 53% patients
**Brain Imaging Findings**
Hydrocephalus: 22 (59%) Diffuse leptomeningeal enhancement: 19 (51%) Stroke: 4 (10%) Normal: 7 (18%)

* Coccidioidomycosis meningitis ** Inter Quartile Range *** cerebrospinal fluid.

**Table 2:** Management and Follow-up of CM * (N = 37).

Characteristics	N (%)
**Antifungal therapy**	
Agent used for initial management of CM	Fluconazole: 33 (89%) Amphotericin 3 (8%)
Fluconazole dose	400 mg: 2 (6%) 600 mg: 3 (9%) 800 mg: 20 (60%) 1000 mg 6 (18%) 1200 mg 2 (6%)
Years on treatment (median with IQR **)	6 [3, 10]
Medication non-adherence to fluconazole	17/33 (51%)
Serologic failure	19/37 (51%)
**Intra cranial Pressure Management**	
VP *** shunt	27 (72%)
Shunt failure and shunt revision	17/27 (62%)
Number of Shunt revisions (range)	1–6
**Follow-up**	
Duration of follow up in months (median with IQR)	48 [24, 84]
Lost to follow-up	18 (48%)
**Death**	6 (16%)

* Coccidioidomycosis meningitis ** Inter-Quartile range *** Ventriculoperitoneal.

**Table 3:** CSF findings at diagnosis of CM * in patients with and without changes of arachnoiditis/syringomyelia.

CSF Findings		Negative (20)	Positive (17)	*p* Value
	Arachnoiditis/Syringomyelia	N (%)	N (%)	
CSF lymphocytic pleocytosis				0.489
No		2 (10.0)	0 (0)	
Yes		18 (90)	17 (100.0)	
CSF Eosinophilia				0.384
No		15 (75.00	12 (70.6)	
Yes		3 (15.0)	5 (29.4)	
CSF culture Positive				0.097
No		14 (70.0)	16 (94.1)	
Yes		16 (30.0)	1 (5.9)	
CSF Cocci IgG Positive				0.999
Yes		19 (95.0)	16 (94.1)	
CSF Cocci IgM Positive				0.981
No		9 (45.0)	8 (47.1)	
Yes		10 (50.0)	8 (47.1)	
CSF Cocci PCR Positive				0.333
No		8 (40.0)	6 (35.3)	
Yes		9 (45.0)	5 (29.4)	
CSF opening pressure, M (SD) **		21.3 (9.9)	34.2 (17.5)	0.034
CSF WBC, M (SD)		1145.1 (3366.7)	505.3 (388.3)	0.442
CSF protein, M (SD)		255.6 (199.5)	877.9 (1225.6)	0.036
CSF glucose, M (SD)		40.3 (19.6)	27.7 (22.9)	0.084

* Coccidioidomycosis meningitis ** Mean (Standard deviation).

## Data Availability

Data supporting the results can be obtained by reaching the corresponding author.

## References

[1] Bays D.J., Thompson G.R. (2021). Coccidioidomycosis. Infect. Dis. Clin. N. Am..

[2] McCotter O.Z., Benedict K., Engelthaler D.M., Komatsu K., Lucas K.D., Mohle-Boetani J.C., Oltean H., Vugia D., Chiller T.M., Cooksey G.L.S. (2019). Update on the Epidemiology of coccidioidomycosis in the United States. Med. Mycol..

[3] Donovan F.M., Shubitz L., Powell D., Orbach M., Frelinger J., Galgiani J.N. (2019). Early Events in Coccidioidomycosis. Clin. Microbiol. Rev..

[4] Rosenstein N.E., Emery K.W., Ben Werner S., Kao A., Johnson R., Rogers D., Vugia D., Reingold A., Talbot R., Plikaytis B.D. (2001). Risk factors for severe pulmonary and disseminated coccidioidomycosis: Kern County, California, 1995–1996. Clin. Infect. Dis..

[5] Stockamp N.W., Thompson G.R. (2016). Coccidioidomycosis. Infect. Dis. Clin. N. Am..

[6] Kelly P.C., Stevens D.A. (1980). Coccidioidal Meningitis. Coccidioidomycosis: A Text.

[7] Johnson R., Ho J., Fowler P., Heidari A. (2018). Coccidioidal Meningitis: A Review on Diagnosis, Treatment, and Management of Complications. Curr. Neurol. Neurosci. Rep..

[8] Wrobel C.J., Chappell E.T., Taylor W. (2001). Clinical presentation, radiological findings, and treatment results of coccidioidomycosis involving the spine: Report on 23 cases. J. Neurosurg..

[9] Sakya S.M., Sakya J.P., Hallan D.R., Warraich I. (2020). Spinal Coccidioidomycosis: A Complication from Medication Noncompliance. Cureus.

[10] Tan L.A., Kasliwal M.K., Nag S., O’Toole J.E., Traynelis V.C. (2014). Rapidly progressive quadriparesis heralding disseminated coccidioidomycosis in an immunocompetent patient. J. Clin. Neurosci..

[11] Jackson N.R., Blair J.E., Ampel N.M. (2019). Central Nervous System Infections Due to Coccidioidomycosis. J. Fungi.

[12] Lammering J.C., Iv M., Gupta N., Pandit R., Patel M.R. (2013). Imaging spectrum of CNS coccidioidomycosis: Prevalence and significance of concurrent brain and spinal disease. AJR Am. J. Roentgenol..

[13] Crete R.N., Gallmann W., Karis J.P., Ross J. (2018). Spinal Coccidioidomycosis: MR Imaging Findings in 41 Patients. AJNR Am. J. Neuroradiol..

[14] Morshed R.A., Lee A.T., Egladyous A., Avalos L.N., Aghi M.K., Theodosopoulos P.V., McDermott M.W., Hervey-Jumper S.L. (2020). Shunt Treatment for Coccidioidomycosis-Related Hydrocephalus: A Single-Center Series. World Neurosurg..

[15] Wright M.H., Denney L.C. (2003). A comprehensive review of spinal arachnoiditis. Orthop. Nurs..

[16] Nadeem S.F., Baig A.N., Tariq Q.U.A., Shamim M.S. (2022). Spinal arachnoiditis and syringomyelia: Review of literature with emphasis on postinfectious inflammation and treatment. Surg. Neurol. Int..

[17] Peng H., Conermann T. (2022). Arachnoiditis. StatPearls [Internet].

[18] Nardone R., Alessandrini F., Tezzon F. (2003). Syringomyelia following Listeria meningoencephalitis: Report of a case. Neurol. Sci..

[19] Nash T.E., O’Connell E.M. (2020). Subarachnoid neurocysticercosis: Emerging concepts and treatment. Curr. Opin. Infect. Dis..

[20] Panackal A.A., Komori M., Kosa P., Khan O., Hammoud D.A., Rosen L.B., Browne S.K., Lin Y.-C., Romm E., Ramaprasad C. (2017). Spinal Arachnoiditis as a Complication of Cryptococcal Meningoencephalitis in Non-HIV Previously Healthy Adults. Clin. Infect. Dis..

[21] Kannapadi N.V., Alomari S.O., Caturegli G., Bydon A., Cho S.-M. (2021). Management of syringomyelia associated with tuberculous meningitis: A case report and systematic review of the literature. J. Clin. Neurosci..

[22] Gupta R., Garg R.K., Jain A., Malhotra H.S., Verma R., Sharma P.K. (2015). Spinal cord and spinal nerve root involvement (myeloradiculopathy) in tuberculous meningitis. Medicine.

[23] Garg R.K., Malhotra H.S., Gupta R. (2015). Spinal cord involvement in tuberculous meningitis. Spinal Cord..

[24] Anderson T.L., Morris J.M., Wald J.T., Kotsenas A.L. (2017). Imaging Appearance of Advanced Chronic Adhesive Arachnoiditis: A Retrospective Review. AJR Am. J. Roentgenol..

[25] Thompson G.R., Ampel N.M., Blair J.E., Donovan F., Fierer J., Galgiani J.N., Heidari A., Johnson R., Shatsky S.A., Uchiyama C.M. (2022). Controversies in the Management of Central Nervous System Coccidioidomycosis. Clin. Infect. Dis..

[26] Galgiani J.N., Ampel N.M., Blair J.E., Catanzaro A., Geertsma F., Hoover S.E., Johnson R.H., Kusne S., Lisse J., MacDonald J.D. (2016). Executive Summary: 2016 Infectious Diseases Society of America (IDSA) Clinical Practice Guideline for the Treatment of Coccidioidomycosis. Clin. Infect. Dis..

[27] Mathisen G., Shelub A., Truong J., Wigen C. (2010). Coccidioidal meningitis: Clinical presentation and management in the fluconazole era. Medicine.

[28] Ho J., Fowler P., Heidari A., Johnson R.H. (2017). Intrathecal Amphotericin B: A 60-Year Experience in Treating Coccidioidal Meningitis. Clin. Infect. Dis..

